# Anthropogenic substrate-borne vibrations impact anuran calling

**DOI:** 10.1038/s41598-019-55639-0

**Published:** 2019-12-19

**Authors:** Valentina Caorsi, Vinicius Guerra, Raíssa Furtado, Diego Llusia, Lívia Roese Miron, Márcio Borges-Martins, Camila Both, Peter M. Narins, Sebastiaan W. F. Meenderink, Rafael Márquez

**Affiliations:** 1 Programa de Pós–Graduação em Biologia Animal, Dep. de Zoologia, Inst. de Biociências, Universidade Federal do Rio Grande do Sul, Av. Bento Gonçalves 9500, Porto Alegre, RS 91540-000 Brazil; 2 Research and Innovation Centre, Fondazione Edmund Mach, via Mach 1, S. Michele all’Adige, 38010, TN Italy; 3 Programa de Pós-Graduação em Ecologia e Manejo de Recursos Naturais, Centro de Ciências Biológicas e da Natureza, Universidade Federal do Acre, Rio Branco, AC Brazil; 4 Programa de Pós-Graduação em Ecologia, Dep. de Ecologia, Inst. de Biociências, Universidade Federal do Rio Grande do Sul, Porto Alegre, CP 15007, RS 91501-970 Brazil; 5 Departamento de Ecología, Terrestrial Ecology Group, Universidad Autónoma de Madrid (UAM), C/Darwin 2, E-28049, Ciudad Universitaria de Cantoblanco, Madrid, Spain; 6 Curso de Ciências Biológicas, Universidade Federal de Santa Maria, Avenida Roraima, n 1000, 97105-900 Santa Maria, RS Brazil; 7 Departamento Interdisciplinar, Universidade Federal do Rio Grande do Sul, Campus Litoral Norte, Av. Tramandaí, 976, 95625-000 Imbé, RS Brazil; 8 Departments of Integrative Biology & Physiology, and Ecology & Evolutionary Biology, University of California Los Angeles, 621 Charles E. Young Drive S., Los Angeles, CA 90095 USA; 9 VA Loma Linda Healthcare System, Loma Linda, CA 92357 USA; 10 Fonoteca Zoológica. Dept. de Biodiversidad y Biología Evolutiva, Museo Nacional de Ciencias Naturales-CSIC, José Gutiérrez Abascal 2, 28006 Madrid, Spain; 11 Laboratório de Herpetologia e Comportamento Animal, Departamento de Ecologia, Inst. de Ciências Biológicas, Universidade Federal de Goiás, Goiânia, GO, Brazil

**Keywords:** Behavioural ecology, Ecology

## Abstract

Anthropogenic disturbance is a major cause of the biodiversity crisis. Nevertheless, the role of anthropogenic substrate vibrations in disrupting animal behavior is poorly understood. Amphibians comprise the terrestrial vertebrates most sensitive to vibrations, and since communication is crucial to their survival and reproduction, they are a suitable model for investigating this timely subject. Playback tests were used to assess the effects of substrate vibrations produced by two sources of anthropogenic activity– road traffic and wind turbines– on the calling activity of a naïve population of terrestrial toads. In their natural habitat, a buried tactile sound transducer was used to emit simulated traffic and wind turbine vibrations, and changes in the toads’ acoustic responses were analyzed by measuring parameters important for reproductive success: call rate, call duration and dominant frequency. Our results showed a significant call rate reduction by males of *Alytes obstetricans* in response to both seismic sources, whereas other parameters remained stable. Since females of several species prefer males with higher call rates, our results suggest that anthropogenically derived substrate-borne vibrations could reduce individual reproductive success. Our study demonstrates a clear negative effect of anthropogenic vibrations on anuran communication, and the urgent need for further investigation in this area.

## Introduction

Environmental pollution (e.g. chemical, noise and light pollution^1,2^ is one of the major causes of the global biodiversity crisis^3^. Among the sources of anthropogenic impact, acoustic pollution is increasingly becoming a threat for natural communities worldwide^4^. This human disturbance has already been shown to negatively affect acoustic communication in many animal groups, such as insects^5^, fish^6^, birds^7^, reptiles^8,9^, amphibians^9–12^ and mammals^13,14^, as well as influencing species persistence and conservation^4^.

Animals can use multiple senses to obtain information about their surroundings^15,16^. Over the past 50 years, scientists have described substrate-borne signaling in a variety of taxa, in the context of sexual selection, territory defense, predator-prey interactions or navigation^17–23^. However, relatively little is known about how animals detect substrate vibrations produced by human activities and how they can be affected by these potentially detrimental cues. One study has shown that anthropogenic substrate-borne vibrations affect the behavior of a hermit crab^24^. On land, it is known that human activities produce seismic vibrations, for instance, induced by traffic road or wind turbines^25,26^, and these anthropogenic vibrations could be sources of mechanical disturbance for animals, but this field is still in its infancy.

Among terrestrial vertebrates, amphibians are the most sensitive to vibrations, and therefore they are a suitable model for assessing potential impacts of human-induced substrate vibrations. The capacity to detect seismic cues is linked to the amphibian inner ear, which comprises three organs known to detect airborne sounds and substrate-borne vibrations: the amphibian papilla, the basilar papilla and the sacculus^27–30^. Despite their seismic sensitivity, vibrational perception and signaling has only been reported for a few species and in limited contexts^22^, e.g. intra-specific signaling^31–33^, prey detection^34^, predator avoidance^35–37^, and detection of environmental cues^38,39^.

Variations in acoustic parameters of the advertisement call, the most commonly emitted call by anuran males, have been linked to species recognition, sexual selection and female choice, which directly affects male reproductive success^40–45^. Hence, changes in calling activity can significantly impact individual fitness and thus species conservation.

Understanding how anthropogenic substrate-borne vibrations may affect animal communication, which directly mediates species reproduction, could help to inform future conservation strategies. To answer the question of the potential effect of anthropogenic ground-borne vibration disturbance on animals, we performed a series of playback tests using two common human-generated activities (road traffic and wind turbine vibrations) to examine behavioral changes in the calling activity of the midwife toad, *A. obstetricans*. The evidence gathered for anurans so far is limited. For instance, species exposed to airborne anthropogenic noise decreased or increased different temporal calling parameters (call rate, call duration) or/and spectral parameters (dominant frequency)^9^. On the other hand, species exposed to rain increased call rate, but the opposite occurred when they were exposed to other natural vibrations such as wind^39^. For the purposes of this study, we hypothesize that human-derived substrate-borne vibrations would exert a negative effect on toad calling activity, i.e., altering the parameters of its calling behavior.

## Results

A total of 26 males of *A. obstetricans* were exposed to vibratory playback stimuli during calling activity. During the playback tests, eight toads showed avoidance behavior between the first and fourth treatment, ceasing calling activity and abandoning their calling site. These animals were excluded from subsequent analyses.

### Call parameters

**Figure 1:**
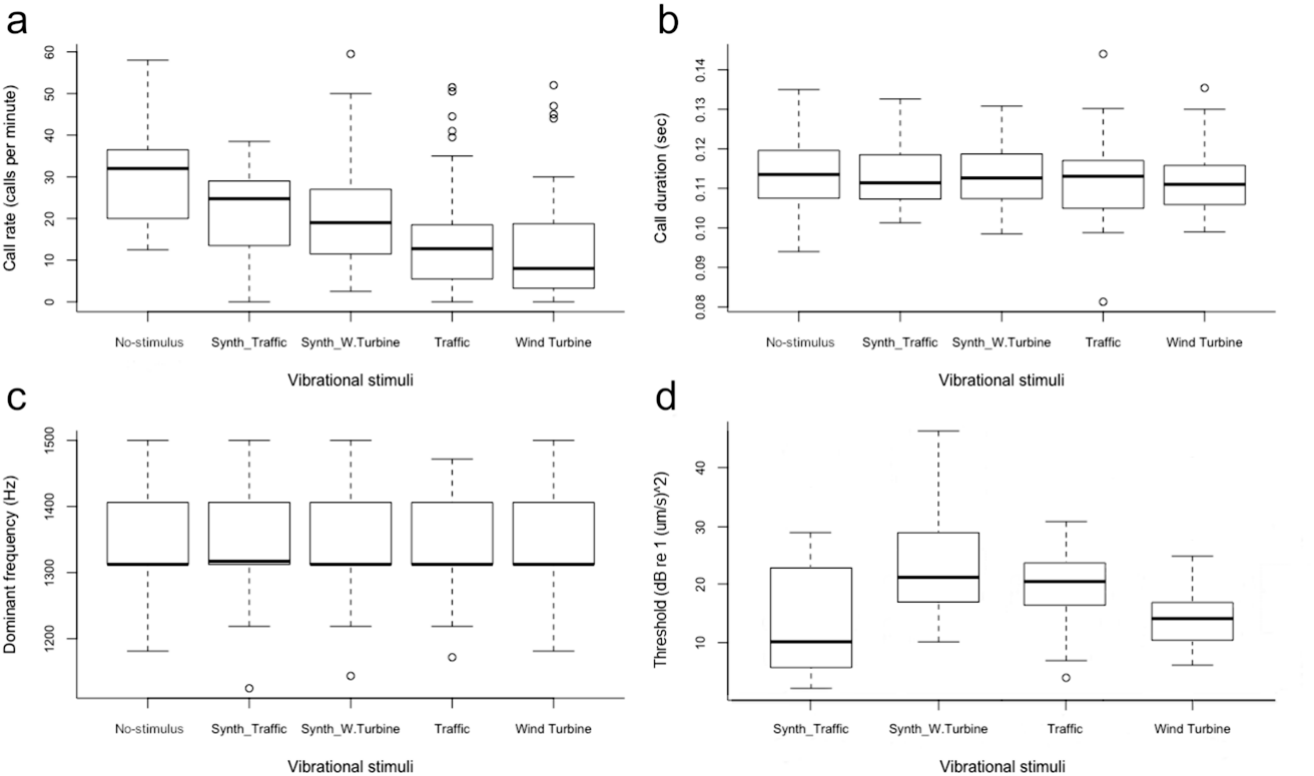
Boxplot showing variation in call parameters during each treatment. (**a**) Call rate, (**b**) call duration and (**c**) dominant frequency and (**d**) power threshold. The box plot displays the median with a center line, a variation of 1^st^ and 3^rd^ quartiles represented by the box, a full range of variation (from min to max) represented by “whiskers” above and below and outliers are represented by small circles.

The calling activity of the common midwife toad was affected by anthropogenic seismic vibrations (Table 1; Fig. 1a). When focal males were exposed to vibratory stimuli, call rate significantly decreased, as shown by the full-null model comparison (n = 18, likelihood-ratio test: *x*^2^ = 24.5, df = 4, p < 0.005; Table 2). Call rate was especially influenced by the recorded traffic and wind turbine stimuli, which caused a mean reduction of 15 and 17.5 calls per min, respectively, while call rate decreased on average by 8 calls per min in response to synthetic stimuli (Table 1; Fig. 1a). Overall, during the no-stimulus periods, calling activity reached maximum rates, up to twice as high as during the original anthropogenic stimuli (Table 1).

**Table 1:** Call parameter variations of the advertisement call emitted by males of midwife toad submitted to traffic and wind turbine vibrations stimuli.

	Call rate (call/min)	Call duration (s)	Dominant frequency (kHz)	Threshold (dB re 1 (um/s)^2)
No-stimulus	30.4 ± 11.2 (12.5–58)	0.114 ± 0.01 (0.09–0.13)	1.34 ± 0.83 (1.2–1.5)	— —
Synthetic T	21.9 ± 10.4 (0–38.5)	0.112 ± 0.008 (0.10–0.13)	1.34 ± 0.96 (1.1–1.5)	13.5 ± 9.8 (1.9–28.7)
Synthetic WT	22.2 ± 15.5 (2.5–59.5)	0.113 ± 0.008 (0.09–0.13)	1.34 ± 0.82 (1.2–1.5)	23.8 ± 9.9 (10.1–46.2)
Traffic	15.05 ± 12.8 (0–51.5)	0.112 ± 0.01 (0.08–0.14)	1.34 ± 0.77 (1.1–1.5)	19.8 ± 7.4 (3.7–45.2)
Wind turbine	12.9 ± 18.8 (0–52)	0.112 ± 0.008 (0.09–0.13)	1.34 ± 0.74 (1.2–1.5)	14 ± 4.95 (6.1–24.7)

Data is given by Mean ± Standard deviation (Range). Synthetic T: synthetic traffic. Synthetic WT: synthetic wind turbine.

**Table 2:** Estimated regression coefficients, standard errors, and confidence intervals for GLMM of call rate in response to vibratory playback stimuli.

	Coefficient	Std. Error	Lower CL	Upper CL
(Intercept)	33.12	4.75	22.82	42.93
Synthetic T	−6.37	2.61	−11.7	−1.12
Synthetic WT	−6.38	4.11	−14.4	1.23
Traffic	−15.45	2.90	−21.36	−9.5
Wind turbine	−18.93	2.68	−24.55	−13.44
Air Temperature	5.5	3.44	−2.33	12.74

CL: confidence limit. Synthetic T: synthetic traffic. Synthetic WT: synthetic wind turbine. No-stimulus was the reference category and air temperature was z-transformed.

In contrast, call duration and dominant frequency remained unaltered during exposure to no-stimulus, synthetic and anthropogenic stimuli (n = 17, likelihood-ratio test: *x*^2^ = 0.28, df = 4, p = 0.99; *x*^2^ = 2.29, df = 4, p = 0.68, respectively; Table 1; Fig. 1b,c). Moreover, baseline acoustic behavior from pre- and post-stimuli periods of the whole experiment showed no differences in any acoustic parameter (likelihood-ratio test: for call rate, *x*^2^ = 0.02, df = 1, p = 0.88; for call duration: *x*^2^ = 0.27, df = 1, p = 0.60, for dominant frequency: *x*^2^ = 2.11, df = 1, p = 0.15). According to the first GLMM, air temperature did not influence call rate during the playback tests (likelihood-ratio test: *x*^2^ = 1.93, df = 1, p = 0.16, in all cases).

### Power threshold

When focal males were exposed to vibratory stimuli, the power threshold necessary to induce a change of calling behavior was different for each stimulus, as shown by the full-null model comparison (n = 15, likelihood-ratio test: *x*^2^ = 25.46, df = 3, p < 0.005; Table 3). The power threshold at the time when males showed a behavioral change in call rate during original noise was higher for traffic than wind farm (Table 1; Fig. 1d), but with a wide range of individual variation. Regarding synthetic stimuli, toads seem to exhibit a higher tolerance to the synthetic wind turbine stimulus, with a spectral peak at 100 Hz compared to that of traffic vibration noise with a spectral peak of 10 Hz (Table 1; Fig. 1d), but again with a wide range of individual variation. In other words, after initiating the playback stimuli, the animals continued calling for longer times (and thus exhibited higher thresholds) in response to the synthetic wind turbine stimulus.

**Table 3:** Estimated regression coefficients, standard errors, and confidence intervals for GLMM of threshold in response to vibratory playback stimuli.

	Coefficient	Std. Error	Lower CL	Upper CL
(Intercept)	−13.34	0.61	−14.56	−12.07
Synthetic WT	2.37	0.64	1.16	3.73
Traffic	1.58	0.53	0.52	2.69
Wind turbine	0.22	0.54	−0.82	1.62
Air Temperature	0.8	0.38	−0.01	1.62

CL: confidence limit. Synthetic WT: synthetic wind turbine. Synthetic Traffic was the reference category and air temperature was z-transformed.

## Discussion

Studies have shown that anthropogenic airborne noise affects acoustic communication e.g.^11,46–49^, but to our knowledge this is the first study to demonstrate the effect of anthropogenic ground-borne vibrations on calling activity of a vertebrate. Playback experiments revealed that anthropogenic vibratory stimuli caused a strong reduction on the calling activity in focal males, decreasing their mean call rate by 50%. Both traffic and wind turbine vibrations had similar impact on calling activity. The comparison of the pre-recorded and synthetic stimuli indicates that the observed responses were triggered by the entire spectrum of the vibrations, rather than by its peak frequency alone. Additionally, the sound power threshold for animals to change their baseline calling activity was lower with playback of naturally recorded wind turbines than with traffic, whereas synthetic traffic stimuli, (spectral peak: 10 Hz) induced a call-rate change at a lower threshold than synthetic wind-turbine playbacks (spectral peak: 100 Hz).

Studies assessing effects of anthropogenic airborne noise on frogs have shown that species coping with this noise use a variety of mechanisms, including short-term adjustment of signal amplitude, timing, duration or even frequencies of their call^9,10^. However, all these reports focused on airborne transmission of signals. In the present study, we found that whereas call duration and frequency were immune to ground-borne vibrations produced by anthropogenic activity, call rate decreased with induced vibrations. Calling activity reached its maximum during no-stimulus, decreased during synthetic emission and decreased further during playback of actual recordings of traffic and wind turbine vibrations.

It is unknown if the studied species, *A. obstetricans*, emit any vibrational signal together with airborne sound as has been described for other anurans^30,33^. However, should this be the case, toads could be susceptible to a substrate-borne masking signal by the vibration noise of the road and wind turbine^50^. A few studies have suggested that anurans may discriminate between different types of vibrational stimuli^35,39^, but it is unknown if midwife toads use this channel for communication.

It is known that two of three sensors in the anuran inner ear are involved in detecting exquisitely low-level substrate-borne vibrations (*sensu*^18,28,51,52^): the amphibian papilla and the sacculus, the latter believed to detect environmental cues^30,34,38^.

Anuran auditory neurons exhibit a clear frequency selectivity that has been characterized in a wide number of frog species by neural tuning curves, or frequency-threshold curves^53,54^. A study tested the sensitivity of the *torus semicircularis* auditory midbrain of *A. obstetricans* to frequencies from 100–5000 Hz. The results showed regions of high sensitivity in the low-frequency range, between approximately 100–500 Hz and, in the high-frequency range, between approximately 1200–2400 Hz. The best thresholds in the lower frequency range reached values of approximately 40 dB SPL, occurring at the lowest audio frequency tested (100 Hz), whereas those in the high-frequency range were between 40 and 50 dB SPL; sensitivity to frequencies below 100 Hz have not been tested in this species^55,56^. Thus, *A. obstetricans* is highly sensitive to very low frequencies, which could explain why the toads decreased calling activity even when their call frequency did not overlap with the noise signal emitted.

Several hypotheses could explain the results found on this study. For example, previous studies with anthropogenic airborne noise, demonstrated that several frog species decreased call rate in response to high levels of interfering noise^11,57^. We found that *A. obstetricans* reduced its call rate during playback of seismic noise, however during the last no-stimulus period of the playback experiment, the animals returned to the base line, pre-stimulus rate. This suggests animals could be adjusting signaling during low-noise periods, which is consistent with noise-avoidance behavior found in airborne sound studies with anurans^11,57^ and other taxa^58,59^.

A previous study mentioned here, reported that frogs apparently discerned between wind and rain seismic cues in the water^39^. Another study showed that two Iberian toad species were able to respond to rainfall-induced vibrations in the soil by emerging to the surface, suggesting that detection of abiotic seismic events might, indeed, be biologically relevant for this group^38^. It is known that call activity not only attracts mates, but also predators, so some anuran species call in choruses or reduce calling to reduce predation risk^60–64^. From a prey perspective, substrate vibrations can signal imminent predation danger, but unlike chemical cues that indicate a general, ongoing level of risk or predator presence in the environment, vibrations indicate current activity of an individual predator. In this sense, the midwife toads could be perceiving the unknown vibration cue as a predator approach and reducing calling activity could reduce the risk. For instance, a study found that vibrational cues alone could elicit substantial levels of early hatching in *Agalychnis callidryas* anticipating a predator attack^35^. The midwife toad’s response of reducing signaling during playback emissions could be related to the association of this interference to a predator. Noise sources that are novel or unpredictable as well as similar to a biologically relevant sound are predicted to elicit responses similar to those associated with predation risk^65^. Hence, reducing or ceasing calling could lower the chances of being located by the predator.

The results from this work could be also related to multimodal interactions among different sensory channels in the frogs. Some species have been shown to respond differently when exposed to multimodal stimuli (e.g.^39^), showing that these animals are not only sensitive to different channels^31^, but also that the interaction between the channels may affect their behavioral response. Furthermore, a given unimodal stimulus presented to the animals may be ineffective compared to a multimodal stimulus^66^.

Regardless of the cause, a reduction in calling rate may have consequences. Several authors have argued that, in addition to ecological impacts of roads, elevated airborne noise levels also impair the ability of animals to effectively communicate during breeding, thereby impacting reproductive success^67–69^. Reproduction usually depends on a female frog’s ability to respond correctly to the advertisement signals of a conspecific male^70^. Therefore, sound localization has obvious fitness consequences for anurans (reviewed in^71–73^). Female anurans exhibit phonotaxis towards male choruses^73^, and noise may impair an individual ability to detect and respond to biologically critical information^74^, affecting mate attraction^69^. It is not known if males of *A. obstetricans* emit seismic signals, or if female choice would be affected by these, but it is known that the female auditory system in this species is highly sensitive to frequencies as low as 100 Hz^55^. Hence, substrate vibrations may represent a source of environmental acoustic information for females. Moreover, it is well known that calling effort is significantly related to female choice in anurans. Females often prefer males with higher call rates^40,42,75^. The same pattern was observed for *A. obstetricans*^45^, which suggests a decrease in call rate could affect female choice and consequently mating success.

## Methods

### Theoretical background

In this work we consider that mechanical waves are divided into *acoustic waves* (purely longitudinal waves in a homogeneous medium, as air, liquid or solid, in which particle motion is in the same direction as energy flow) and *surface-borne waves* (that occur at boundaries between different media, where energy is transferred from one medium to the other, and in which substrate particles oscillate in a plane perpendicular to the direction of energy flow (e.g.^17^). The focus of this study– vibrations caused by anthropogenic sources– are surface-borne vibrations of the Rayleigh wave type (with particle motion in the vertical direction, perpendicular to the direction of energy flow^17,21,76^). We have also used the terms ground-borne vibration or seismic vibration, which refer to the waves carried in soil or sand^21^.

### Study area and species

Playback tests were conducted in Lago de la Cueva (43°3′N, 6°6′W, 1550 m.a.s.l.) in Somiedo Natural Park, Asturias, Spain. Male midwife toads (*A. obstetricans*) call from the ground below rocks or in holes^77,78^. Their simple advertisement calls have been described^79,80^. Experiments took place from 12–25 June 2017, during the breeding season, during clear sky nights (Temp 13.6–25.6 °C).

### Vibratory stimuli

**Figure 2:**
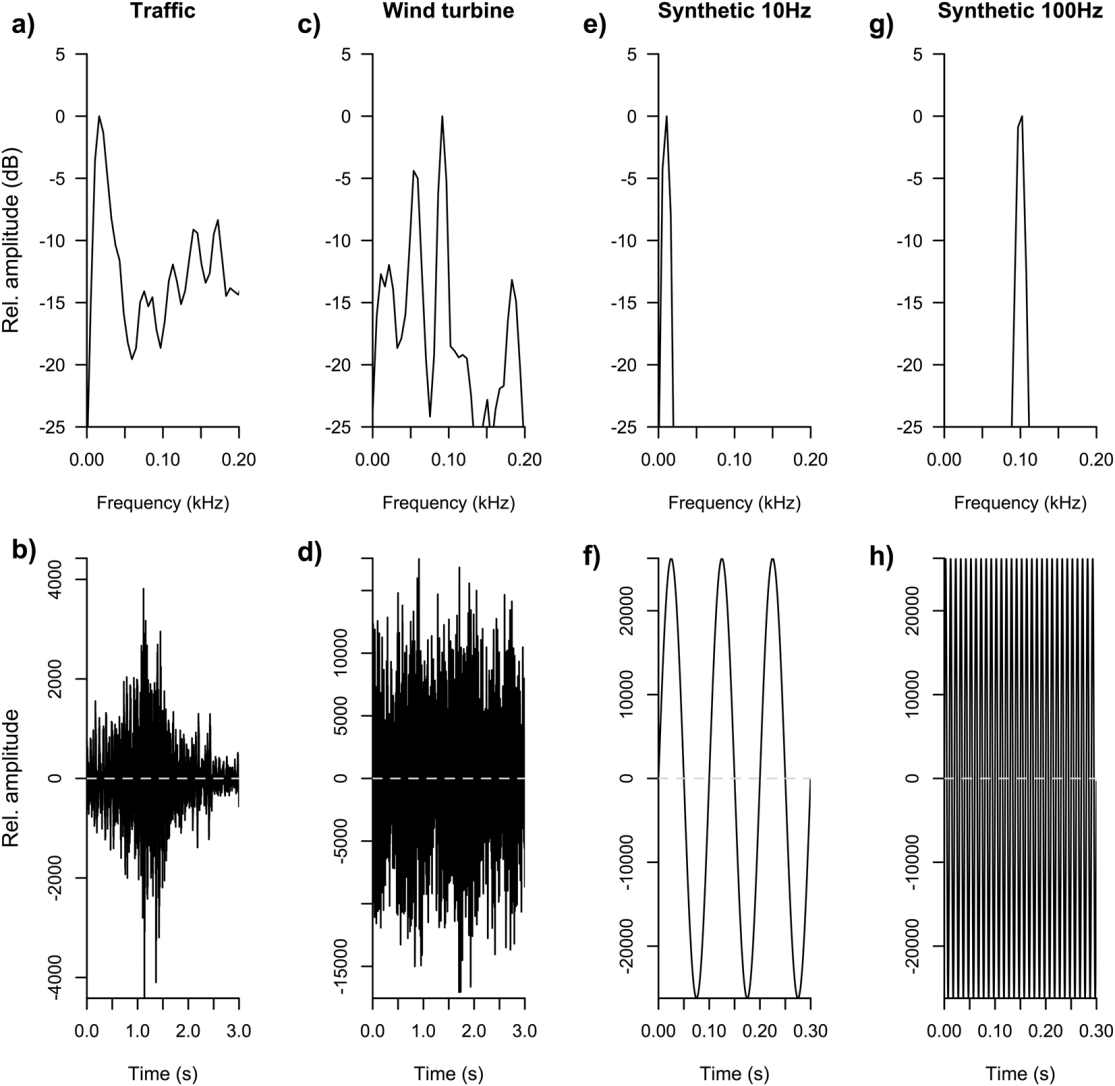
Vibration stimuli used in the study. Amplitude spectrum (above) and waveform (below) of the road traffic and wind turbine seismic vibrations recorded and synthetic stimuli constructed.

We constructed a set of five playback vibratory stimuli (Fig. 2): (i) road traffic, (ii) a wind turbine, (iii) a synthetic imitation of road traffic (iv) a synthetic imitation of a wind turbine, and (v) no-stimulus (control). Three different copies of the original stimuli of road traffic and wind turbine were prepared to provide replicates of the playback stimuli. Synthetic imitations of original vibrations were used to control the acoustic properties of the stimuli, in order to test behavioral responses to particular spectral components of anthropogenic vibrations. The no-stimulus period was used as control to account for absence of anthropogenic vibrations. Each experiment consisted, therefore, of a total of nine playback conditions. None of the focal animals had been previously exposed to any of the playback stimuli.

Playback stimuli of traffic road and wind turbine vibrations were recorded on 7 June, 2017 at Fuencarral-El Pardo road (40°30′16.01 N, 3°45′06.62 W, 663 m.a.s.l., Madrid) and on 9 June, 2017 at Canredondo, (40°48′11 N, 2°32′22 W, 1210 m.a.s.l., Castilla-La Mancha), respectively. The maximum speed limit on this two-lane road was 60 km/h and during recordings the car flow was ca. 5 cars/min. The wind turbine had three 83-m blades (Gamesa G83/2000) and was operating with a wind speed of 10–12 km/h on the recording day. In each location we recorded for approximately 1 hour. For the recordings, we used a geophone (OYO–One, Oyo-Geospace), placed 4 m from the source (either the roadside or the wind turbine), connected to a custom-built amplifier, which fed a digital recorder (Sound Devices, model 744 T). Recordings were made using a sampling rate of 48000 Hz and 16-bit resolution, and saved in an uncompressed .wav format.

Recordings of the two vibrational signals were edited in a laptop computer (MacBook Pro Intel Core i7) using Audacity 2.1.1 audio edition. Segments of 5–10 s judged to have spectra representative of each noise type were selected and pasted to create noise segments up to 120 s in duration. To create synthetic imitations, FFTs of the traffic and wind turbine vibration recordings were calculated (1,024 Audacity 2.0.2 and Raven Pro 2.5 software; Fig. 2a–d). The frequency content of the traffic and wind turbine stimuli ranged from near 0 Hz to 500 Hz, with peak frequencies at 10 Hz and 100 Hz, respectively, in accord with previous literature^26^. Thus, we constructed two synthetic stimuli by generating a single sinusoid at the dominant frequency of each vibratory source (10 Hz for road traffic and 100 Hz for wind turbine; Fig. 2e–h). Thus, the synthetic stimuli mimicked the central spectral component of the natural vibrations but lacked other secondary elements observed in the field recordings.

**Figure 3:**
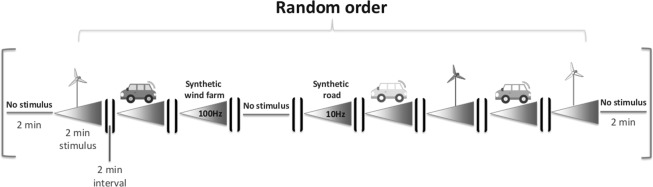
Playback scheme showing the 38-min playback presented to each animal. It contained a total of nine fragments of five different stimuli. Triangles indicate the increase of amplitude from 0–100% of the vibration emission within the 2-min treatment.

Playback tests followed the A-B-A protocol^81–83^. Each of the nine playback tracks containing a single treatment or stimulus lasted 2 min and was preceded and followed by a 2-min interval of no-stimulus to allow the animal to return to its baseline behavior. Thus, the total test duration was 38 min/animal. To standardize stimulus amplitudes, all were peak normalized. Moreover, each stimulus track was modified by applying a linear ‘fade in’ amplitude filter^38^ from 0 to 100% over 2 min to expose the focal individuals to a monotonically increasing vibration throughout each playback presentation (Fig. 3).

### Experimental procedures

**Figure 4:**
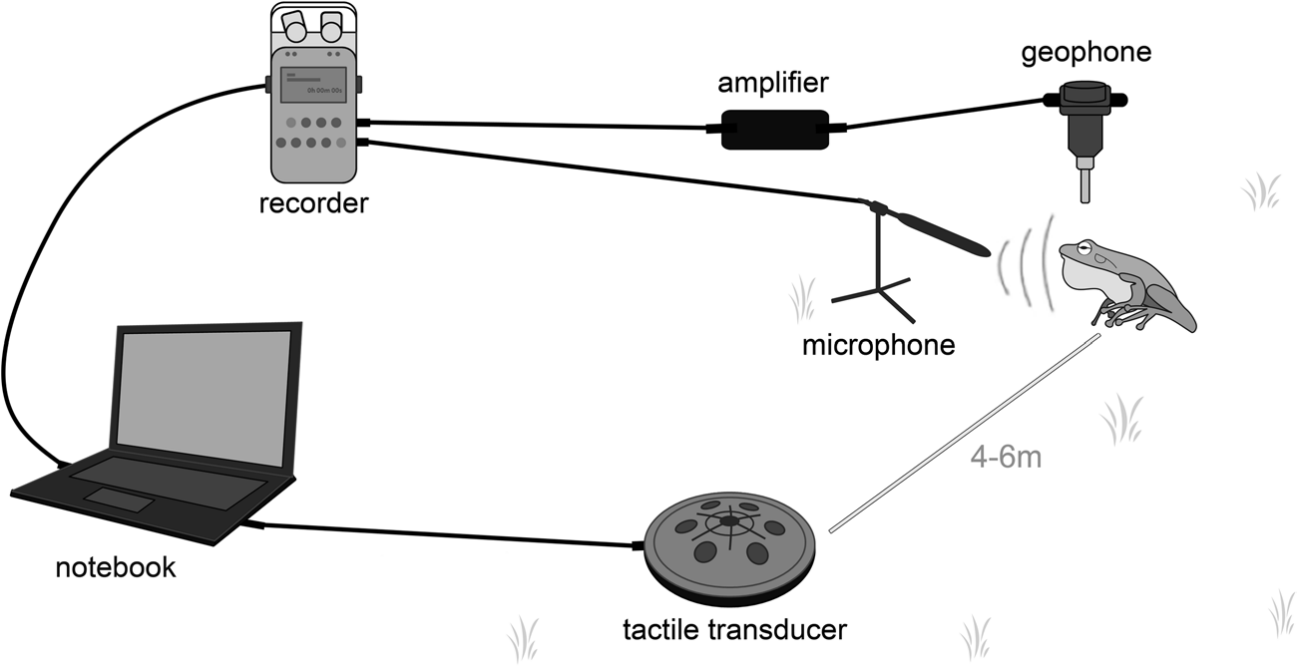
Equipment setup used for playback experiments and recording of seismic vibrations.

Experiments were carried out during 13 days from sunset until dawn, when male toads were calling intensively. Prior to a playback experiment, a geophone and a microphone were placed at a distance of 20–30 cm from the focal animal, the tactile transducer was buried 5–10 cm below ground between 4–6 m away from the male, all lights were extinguished, and observers moved at least 6 m away from the focal animal, and remained motionless to allow the focal individual to resume calling (Fig. 4).

Playback vibrations were generated using Audacity 2.0.2 software on a MacBook Air computer. The audio output from the computer was fed to an amplifier (Kenwood KAC-5205; frequency range: 5–50,000 Hz), connected to a tactile sound transducer (Clark Synthesis, Platinum model, frequency range 5–17,000 Hz). The output signal was calibrated by setting the audio output of the computer to a fixed level (−12 dB) and using the amplifier fixed level and bridged output. The vibrations generated by the tactile sound transducer using these settings were monitored with a geophone (Oyo-One, Oyo-Geospace). The output of the geophone was amplified by 60 dB and connected to the line input of the digital recorder (Sound Devices, model 744 T). At the beginning of the experiment set, a 1-s wind-turbine test stimulus was emitted and the distance of the tactile transducer was adjusted to ensure that animals were receiving signals of equal amplitude.

During the tests, the order of stimuli was randomized for each animal and air temperature and relative humidity were monitored every 15 min using an environmental outdoor data logger (HOBO 64 K Pendant^®^Temperature/Alarm and HOBO Pro-V2, respectively). Since previous studies have documented call site fidelity in males of *A. obstetricans*^84^, we marked the location of each focal male and moved 2–15 m away along the lakeshore to prevent recording duplication.

All the equipment was tested in the field using pure tones from 10–300 Hz (generated with Audacity) and synthetic tone amplitudes were systematically equalized to compensate for differences in equipment properties.

### Ethical and legal permits

Access and study permits were granted by the Somiedo Natural Park administration, Principado de Asturias, Spain.

### Acoustic analyses

The primary acoustic parameters of the advertisement calls of each male were measured with Raven Pro v. 1.4 software: call rate ([number of calls – 1]/min); call duration (s); and dominant frequency (Hz). Temporal parameters were measured from oscillograms, while spectral parameters were measured from spectrograms created using a Hann window, a window length of 512 points and 50% overlap. For these measurements, we randomly selected ten calls for each treatment for each toad.

To obtain a measure of the amount of vibratory power that triggered a behavioral response, we calculated the power (Matlab’s *bandpower.m*; bandwidth: 0–500 Hz) in the geophone’s signal over a two-second window immediately preceding the time at which the animal first changed its call rate in response to the stimulus. The background noise measured before the stimulus emission was subtracted from that value. In order to facilitate comparisons of the data obtained, the original values measured in Volt^2, were transformed to dB re. 1 (µm/s)^2. The values corresponding to the behavioral thresholds measured are within the range of amplitudes of the vibrational stimuli used in this study. Therefore, the values are not directly comparable to traffic power values from studies of the effect of anthropogenic noise in airborne on animals^9^ found in the literature.

### Statistical analyses

General linear mixed-effects models (GLMM^85^) were used to test the effect of traffic and wind turbine vibrations on calling activity of the focal individuals. First, a GLMM for each acoustic parameter (call rate, call duration, dominant frequency and power threshold) was set using Gaussian error structure and identity link function to search for the relationship between these parameters and the vibratory playback stimuli. In these models, *type of stimuli* was included as a fixed factor, *air temperature* as a covariate, and *recording day, individual* and *track* as random factors. In the case of power threshold, the data was log transformed. Second, to test whether call parameters varied between the silent periods before and after the exposition to stimuli, similar GLMMs were set using 1 min of no-stimulus prior to and following the playback experiment. In these models, *period* (*pre- and post-test*) was included as a fixed factor, *air temperature* as a covariate and *recording day* and *individual* as random factors. Additionally, the required random slopes (all except that of type of treatment within track, n = 5) were included in the models in order to keep type I error at the nominal level of 5%^86,87^. The random structure of one model (power threshold) was simplified to achieve model convergence. Correlation parameters between random intercepts and random slope terms were also added when model convergence was not compromised. To reduce model complexity, interaction terms between fixed factors were excluded.

Model inference and the effect of individual predictors were established by full-null model comparisons^38^. Visual inspection of Q-Q plots and residuals plotted against fitted values revealed no obvious deviation from the canonical assumptions of normally distributed and homogenous model residuals. Colinearity issues were absent from the models according to the Variance Inflation Factor (VIF < 1.64, in all cases), estimated with the function vif of the R-package^88^ with a standard linear model excluding random effects. Confidence intervals of model coefficients were computed through 1000 bootstrap iterations using the function bootMer of the R-package lme4^89^. GLMMs were fitted in R^90^ using the functions lmer of the package lme4^89^.

### Statement

I hereby certify that all methods were carried out in accordance with relevant guidelines and regulations.

## Supplementary information

Supplementary Information

## Data Availability

The data used for the analysis is available for access (Supplementary Table S1).
