# Supplementary material for: Anthropogenic substrate-borne vibrations impact anuran calling
Source: Sci Rep. 2019 Dec 19;9:19456. doi: 10.1038/s41598-019-55639-0 (PMC6923410; doi:10.1038/s41598-019-55639-0)
Supplement: Supplementary file 1 — Supplementary Information [file 41598_2019_55639_MOESM1_ESM.pdf]

Anthropogenic substrate-borne vibrations impact anuran calling

Valentina Caorsi , Vinicius Guerra, Raíssa Furtado, Diego Llusia, Lívia Roese Miron, Márcio Borges-Martins, Camila Both, Peter M. Narins, Sebastiaan W.F. Meenderink, Rafael Márquez

Supplementary Table S1 – Data from the advertisement calls of *Alytes obstetricans* collected during playback experiments and used for analysis. The rows contain the **Individual** recorded; the **Day** of the month of June 2017; the temperature (**Temp**) of the air (ºC) during each recording; the **Type** of stimuli played: Traffic, Synthetic Traffic, Wind Turbine, Synthetic Wind Turbine and Silence; average **Call rate** (number of calls/minute) of each individual during each type of stimulus; average **Call duration** (sec) for the calls emitted during each stimulus); average **D**ominant **frequency** (Hz) for the calls during each stimulus; **Threshold** (dB re 1 (um/s)^2) of change in baseline calling activity during each stimulus. NA stands for the missing data.

Individual	Day	Temp	Type	Call rate	Call duration	D. frequency	Threshold
2	13	15	Wind turbine	24.5	0.108	1312.5	6.09
2	13	15	Silence	42.5	0.1194	1359.35	NA
2	13	15	Synthetic Wind turbine	30	0.1149	1406.2	NA
2	13	15	Synthetic Traffic	25.5	0.119	1406.2	NA
2	13	15	Traffic	12	0.1134	1406.2	18.45
2	13	15	Wind turbine	14	0.1147	1406.2	6.25
2	13	15	Wind turbine	24.5	0.1168	1406.2	15
2	13	15	Traffic	14.5	0.1148	1406.2	17
2	13	15	Traffic	2.5	0.1178	1406.2	20.4
3	13	15	Wind turbine	6.5	0.1252	1312.5	NA
3	13	15	Synthetic Traffic	24	0.1326	1312.5	NA
3	13	15	Traffic	15.5	0.1294	1312.5	NA
3	13	15	Silence	26	0.135	1312.5	NA
3	13	15	Synthetic Wind turbine	16.5	0.1308	1312.5	NA
3	13	15	Traffic	18.5	0.1302	1312.5	NA
3	13	15	Wind turbine	16.5	0.1354	1312.5	NA
3	13	15	Wind turbine	2	0.12825	1312.5	NA
3	13	15	Traffic	4.5	0.1236	1302.1	NA
4	13	15	Wind turbine	0.5	0.121	1406.2	NA
4	13	15	Traffic	0	NA	NA	NA
4	13	15	Synthetic Traffic	13	0.1127	1406.2	NA
4	13	15	Synthetic Wind turbine	4	0.112625	1359.35	NA
4	13	15	Silence	20	0.1241	1406.2	NA

Individual	Day	Temp	Type	Call rate	Call duration	D. frequency	Threshold
4	13	15	Wind turbine	0	NA	NA	NA
4	13	15	Traffic	5	0.1128	1406.2	NA
4	13	15	Traffic	0	NA	NA	NA
4	13	15	Wind turbine	2.5	0.1106	1406.2	NA
5	14	16.72	Silence	32	0.106	1275.02	NA
5	14	16.72	Wind turbine	7	0.1099	1303.13	24.7
5	14	15.39	Synthetic Wind turbine	50	0.108	1303.13	NA
5	14	15.39	Synthetic Traffic	29.5	0.108	1312.5	5.5
5	14	15.39	Traffic	14.5	0.1083	1312.5	23.9
5	14	15.39	Wind turbine	2	0.1015	1312.5	11.7
5	14	14.72	Traffic	11	0.1048	1312.5	27.4
5	14	14.72	Traffic	26.5	0.1068	1312.5	16.17
5	14	14.72	Wind turbine	1.5	0.1023	1312.5	21.38
7	15	14.96	Traffic	22	0.105	1406.2	22.34
7	15	14.96	Synthetic Wind turbine	19.5	0.1062	1406.2	27.53
7	15	14.96	Silence	27	0.109	1406.2	NA
7	15	13.69	Wind turbine	12	0.1134	1406.2	7.9
7	15	13.69	Synthetic Traffic	14	0.1101	1406.2	23.68
7	15	13.69	Traffic	18	0.1042	1406.2	22.38
7	15	13.69	Traffic	25	0.1059	1406.2	NA
7	15	16.68	Wind turbine	7	0.1047	1406.2	15.11
7	15	16.68	Wind turbine	18.5	0.1072	1406.2	NA
8	15	13.57	Traffic	3	0.1162	1312.5	15.36
8	15	13.57	Synthetic Traffic	27.5	0.1136	1312.5	21.73
8	15	13.57	Wind turbine	10.5	0.1153	1312.5	10.67
8	15	13.57	Synthetic Wind turbine	11.5	0.1195	1303.13	30.71
8	15	13.57	Silence	18.5	0.1135	1312.5	NA
8	15	13.57	Wind turbine	6.5	0.1149	1312.5	18.84
8	15	13.57	Traffic	4	0.1143	1312.5	21.11
8	15	13.57	Wind turbine	7.5	0.1148	1312.5	10.87
8	15	13.57	Traffic	8	0.1158	1312.5	25.09
9	15	15.1	Wind turbine	3	0.1247	1312.5	NA
9	15	13.85	Synthetic Traffic	35	0.1073	1321.87	NA
9	15	13.85	Traffic	0.5	0.144	1406.2	NA
9	15	13.85	Silence	41	0.1098	1368.72	NA
9	15	13.85	Synthetic Wind turbine	44	0.1079	1359.35	NA
9	15	16.6	Traffic	39.5	0.107	1387.46	NA
9	15	16.6	Traffic	51.5	0.1082	1368.72	NA
9	15	16.6	Wind turbine	47	0.1059	1406.2	NA
9	15	16.6	Wind turbine	52	0.1075	1406.2	NA
10	15	15.62	Traffic	18	0.1196	1415.58	23.86
10	15	15.62	Wind turbine	19	0.1153	1500	15.22
10	15	15.62	Synthetic Wind turbine	21.5	0.1227	1415.58	46.19
10	15	15.62	Silence	36.5	0.1278	1453.1	NA
10	15	14.529	Synthetic Traffic	28.5	0.1197	1471.86	1.88

Individual	Day	Temp	Type	Call rate	Call duration	D. frequency	Threshold
10	15	14.529	Traffic	11.5	0.1194	1500	24.36
10	15	14.529	Wind turbine	22	0.1202	1500	15.44
10	15	14.529	Wind turbine	20.5	0.1242	1500	17.23
10	15	14.529	Traffic	NA	NA	NA	NA
12	16	19.24	Traffic	7	0.1098	1237.54	27.36
12	16	19.24	Wind turbine	4	0.1088	1312.5	18.29
12	16	19.86	Synthetic Traffic	0	NA	NA	3.85
12	16	19.86	Synthetic Wind turbine	22	0.1113	1312.5	21.17
12	16	19.86	Silence	19	0.1121	1293.76	NA
12	16	19.88	Traffic	9	0.1126	1312.5	20.89
12	16	19.88	Traffic	11	0.1179	1312.5	30.65
12	16	19.88	Wind turbine	4.5	0.1093	1312.5	23.7
12	16	20.2	Wind turbine	18	0.1096	1312.5	13.7
13	16	19.2	Traffic	2.5	0.1144	1500	23.26
13	16	19.2	Synthetic Traffic	8.5	0.1139	1500	28.74
13	16	19.2	Wind turbine	0	NA	NA	11.27
13	16	19.2	Synthetic Wind turbine	2.5	0.1166	1500	25.20
13	16	19.2	Silence	12.5	0.1153	1471.86	NA
13	16	19.27	Traffic	5	0.1198	1406.2	26.6
13	16	19.27	Traffic	0.5	0.121	1406.2	13.03
13	16	19.27	Wind turbine	0	NA	NA	17.7
13	16	19.46	Wind turbine	0	NA	NA	16.78
15	17	22.92	Wind turbine	44	0.13	1228.17	NA
15	17	22.92	Silence	33.5	0.1196	1171.9	NA
15	17	22.729	Traffic	44.5	0.1227	1228.17	26.76
15	17	22.729	Synthetic Traffic	NA	NA	NA	NA
15	17	22.729	Synthetic Wind turbine	59.5	0.1209	1218.8	16.94
15	17	22.369	Traffic	41	0.1132	1228.17	19.11
15	17	22.369	Traffic	50.5	0.1201	1218.8	NA
15	17	22.369	Wind turbine	30	0.1155	1218.8	21.21
15	17	22.298	Wind turbine	45	0.1217	1228.17	NA
17	17	21.819	Wind turbine	8	0.1149	1312.5	16.83
17	17	21.819	Silence	34	0.1228	1340.61	NA
17	17	21.079	Synthetic Wind turbine	17	0.1187	1321.87	28.79
17	17	21.079	Synthetic Traffic	NA	NA	NA	NA
17	17	21.079	Traffic	10.5	0.1161	1312.5	45.18
17	17	20.722	Wind turbine	6	0.1114	1312.5	16.31
17	17	20.722	Wind turbine	8	0.1115	1387.46	15.11
17	17	20.722	Traffic	16.5	0.116	1378.09	21.48
17	17	20.889	Traffic	14.5	0.1159	1368.72	NA
18	18	22.25	Silence	58	NA	NA	NA
18	18	22.25	Wind turbine	24.5	NA	NA	13.76
18	18	22.25	Traffic	35	NA	NA	21.74
18	18	22.633	Synthetic Wind turbine	18.5	NA	NA	35.34
18	18	22.633	Synthetic Traffic	38.5	NA	NA	7.16

Individual	Day	Temp	Type	Call rate	Call duration	D. frequency	Threshold
18	18	22.633	Traffic	NA	NA	NA	NA
18	18	22.896	Traffic	30.5	NA	NA	7.01
18	18	22.896	Wind turbine	3.5	NA	NA	9.33
18	18	22.896	Wind turbine	NA	NA	NA	NA
19	18	19.936	Traffic	2.5	0.1032	1218.8	18.67
19	18	20.079	Wind turbine	1	0.104	1218.8	7.79
19	18	20.079	Silence	14	0.094	1218.8	NA
19	18	20.079	Synthetic Traffic	12.5	0.1013	1218.8	10.10
19	18	19.793	Synthetic Wind turbine	11	0.1074	1181.28	14.19
19	18	19.793	Wind turbine	2.5	0.1018	1181.28	NA
19	18	19.793	Traffic	6	0.1049	1143.76	14.57
19	18	19.532	Wind turbine	0.5	0.107	1218.8	NA
19	18	19.532	Traffic	1	0.1105	1218.8	16.56
21	19	21.056	Wind turbine	24.5	0.1019	1312.5	14.05
21	19	21.318	Traffic	24	0.1018	1312.5	NA
21	19	21.318	Silence	36.5	0.1061	1312.5	NA
21	19	21.318	Synthetic Wind turbine	27	0.103	1312.5	15.23
21	19	21.628	Wind turbine	15.5	0.099	1312.5	6.68
21	19	21.628	Traffic	18.5	0.0999	1312.5	9.84
21	19	21.628	Synthetic Traffic	28	0.1045	1312.5	NA
21	19	22.011	Wind turbine	11	0.1012	1312.5	11.63
21	19	22.011	Traffic	9	0.0813	1265.65	3.69
24	21	18.652	Wind turbine	25	0.1118	1349.98	9.25
24	21	18.652	Silence	35.5	0.116	1312.5	NA
24	21	18.652	Traffic	16.5	0.1129	1396.83	19.81
24	21	18.295	Traffic	18.5	0.1135	1368.72	21.32
24	21	18.295	Synthetic Wind turbine	24	0.1175	1312.5	10.11
24	21	18.295	Wind turbine	12	0.1158	1406.2	7.45
24	21	19.032	Synthetic Traffic	16.5	0.1185	1331.24	26.23
24	21	19.032	Traffic	14	0.1163	1378.09	21.24
24	21	18.628	Wind turbine	4.5	0.1174	1343.73	NA
26	22	15.294	Traffic	10	0.101	1406.2	6.87
26	22	15.294	Synthetic Traffic	20.5	0.108	1125	13.47
26	22	15.151	Silence	29	0.0988	1471.86	NA
26	22	15.151	Synthetic Wind turbine	16	0.0985	1481.24	19.93
26	22	15.151	Wind turbine	NA	NA	NA	NA
26	22	14.697	Traffic	11.5	0.0988	1481.24	11.01
26	22	14.697	Wind turbine	12.5	0.1009	1424.96	10.31
26	22	14.697	Traffic	13.5	0.0997	1434.34	11.58
26	22	14.409	Wind turbine	14	0.1022	1415.58	13.81
27	22	14.84	Synthetic Traffic	30	0.1073	1312.5	5.71
27	22	14.84	Wind turbine	6.5	0.1075	1312.5	10.1
27	22	14.84	Synthetic Wind turbine	6	0.1056	1312.5	17.89
27	22	15.199	Traffic	6.5	0.1034	1312.5	18.99
27	22	15.199	Silence	32	0.1075	1312.5	NA

Individual	Day	Temp	Type	Call rate	Call duration	D. frequency	Threshold
27	22	15.676	Wind turbine	8	0.1058	1312.5	21.88
27	22	15.676	Traffic	14.5	0.1038	1312.5	16.91
27	22	15.676	Traffic	13.5	0.1061	1312.5	19.93
27	22	16.082	Wind turbine	13.5	0.1061	1321.87	15.55
